# Interleukin levels in neovascular age-related macular degeneration: evaluation of morphological and functional progression over 5 years

**DOI:** 10.1007/s00417-022-05635-4

**Published:** 2022-04-12

**Authors:** Michelle Prasuhn, Khaled Nassar, Aysegül Tura, Mahdy Ranjbar

**Affiliations:** Department of Ophthalmology, University Hospital Schleswig-Holstein, University of Lübeck, Ratzeburger Allee 160, 23538 Lübeck, Germany


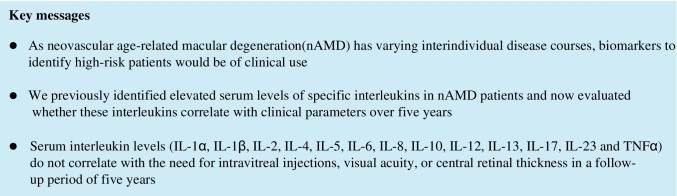


Dear editor,

Neovascular age-related macular degeneration (nAMD) is a major cause of vision loss world-wide and becomes more and more common due to the demographic change in developed countries. The high prevalence and severe personal burden for affected patients generate the urge to gain a deeper understanding of the disease entity and to optimize patient care. For both aspects, we analyzed specific interleukin (IL) levels in sera of nAMD patients several years ago [1]. Herein, we would like to report on our follow-up analyses of that cohort. As previously reported, we collected serum samples from 30 patients with nAMD and 15 age-matched healthy donors, both without systemic inflammatory diseases. For this study, we included the previously described nAMD patients who regularly attended follow-up visits over 5 years in our clinic, while exclusion criteria included other ocular neovascular diseases, glaucoma, chronic uveitis, intra- or extra-ocular tumors, patients suffering from rheumatoid arthritis, inflammatory bowel disease, spondylarthropathy synovitis, tuberculosis, and malignant tumor (for details of the chosen cohort, see reference [1]). We carried out ELISAs to determine serum levels of IL-1α, IL-1β, IL-2, IL-4, IL-5, IL-6, IL-8, IL-10, IL-12, IL-13, IL-17, IL-23, and TNFα and demonstrated significantly elevated concentrations of IL-1α, IL-1β, IL-4, IL-5, IL-10, IL-13, IL-17, and TNFα in nAMD patients compared to the control group.

Our current analyses aimed at determining whether these IL levels are associated with different disease progressions. Thus, we retrospectively evaluated clinical data of our patient collective over 5 years and we were able to generate complete follow-up datasets of 24 patients (Table 1). Endpoints of our analyses included a number of intravitreal injections (IVI) over the 5 years, change in best-corrected visual acuity (BCVA), and change in central retinal thickness (CRT). A partial correlation between the clinical data and the serum levels (IBM SPSS version 27.0) was done and results were visualized using a heatmap (GraphPad Prism version 9.0). Confounding variables were age and sex as they impact the clinical and laboratory values.

**Table 1:** Epidemiological and clinical data of included patients with complete datasets over 5 years. Data are reported as median (minimum; maximum)

	*n* = *24*
Sex (f:m)	17:7
Age (years)	77.5 (65; 84)
Change in visual acuity over 5 years (logMar)	0.4 (0.1; − 1.3)
Change in central retinal thickness over 5 years (µm)	− 50.5 (− 230; 261)
Total number of intravitreal injections in 5 years	22.5 (3; 43)

**Fig. 1:**
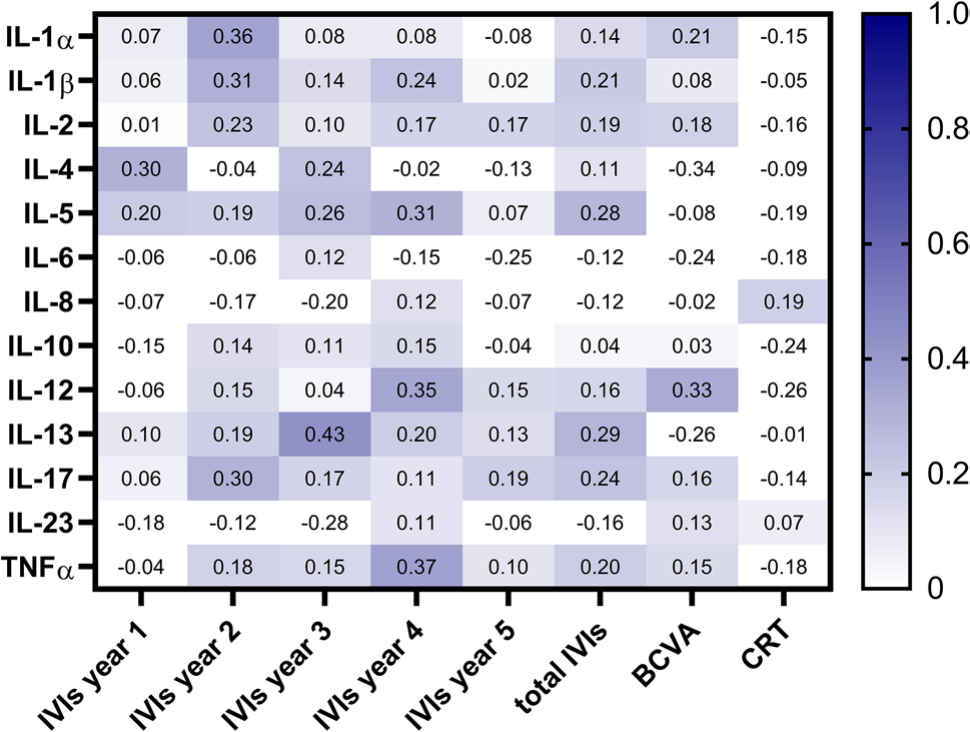
Heatmap visualizing the correlation coefficients of partial correlations of serum levels (left) and clinical endpoints (below), correcting for age and sex. The numbers represent correlation coefficients as results of the partial correlation and are visualized in shades of blue: the darker the blue, the stronger the correlation. BCVA: best-corrected visual acuity; CRT: central retinal thickness; IL: interleukin; IVI: intravitreal injection; TNFα: tumor necrosis factor α

Results are displayed in Fig. 1. It can be seen that serum levels of the analyzed interleukins do correspond to neither the number of IVIs needed, the change in BCVA, nor CRT changes.

Patients with nAMD regularly receive IVIs with anti-vascular endothelial growth factor therapy (anti-VEGF). Even though adverse events are rare, endophthalmitis and severe vision loss or even blindness can occur. Additionally, the regular visit rate is a high burden for elderly patients not only financially and socially but also psychologically. A wide variability concerning interindividual disease progression in nAMD causes several patients to be visited too frequently or not frequently enough and therefore to be over- or undertreated. Consequently, there is a high need for markers that allow us to identify patients who need to be followed up more regularly or who can gain more individual freedom by expanding clinical visit intervals.

Undoubtedly, the relevance to identify specific biomarkers like interleukins is of high interest to the research community, as many study groups started different attempts in various study designs.

More and more evidence underlines that immunological processes play an important role in the pathogenesis of nAMD [2–4]. Therefore, and having our previous results in mind, we found our approach to be promising to gain more insights into the disease current in connection with inflammatory changes. Our retrospective analyses were not able to detect correlations between specific interleukins and our clinical parameters. However, as reported in our previous manuscript, altered interleukin levels are present in sera of nAMD patients, so longitudinal prospective studies with larger cohorts are still a reasonable approach to look at this issue more in detail. The clinical course is one of the most important questions to address, as nAMD lacks specific and easily available prognostic markers. This could help us in clinical routine to identify patients who need to be followed up more regularly, as nAMD shows highly variable interindividual courses.
